# Comprehensive antiphospholipid antibody profiling and unsupervised immune phenotyping in fetal growth restriction

**DOI:** 10.3389/fimmu.2026.1845168

**Published:** 2026-06-03

**Authors:** Siyin Li, Xiaofang Shu, Juan Shi, Jinmei Zou, Kai Wang

**Affiliations:** 1 Department of Rheumatology and Immunology, Mianyang Central Hospital, School of Medicine, University of Electronic Science and Technology of China, Mianyang, China; 2 Department of Obstetrics and Gynecology, Mianyang Central Hospital, School of Medicine, University of Electronic Science and Technology of China, Mianyang, China; 3 Department of Rheumatology and Immunology, The Affiliated Huaian No.1 People’s Hospital of Nanjing Medical University, Huaian, China

**Keywords:** antiphospholipid antibodies, fetal growth restriction, hierarchical clustering, non-criteria antibodies, pregnancy outcome, seronegative antiphospholipid syndrome

## Abstract

**Background:**

Non-criteria antiphospholipid antibodies (aPLs) may be associated with adverse obstetric outcomes in patients who do not meet conventional antiphospholipid syndrome (APS) classification criteria. Their clinical relevance in fetal growth restriction (FGR) remains incompletely defined.

**Methods:**

This retrospective cohort study included 104 pregnant women with FGR or related adverse obstetric presentations who underwent comprehensive testing for 26 solid-phase aPL markers, together with lupus anticoagulant assessment. Criteria-aPL positivity was defined as positivity for lupus anticoagulant, anticardiolipin IgG/IgM, or anti-β2-glycoprotein I IgG/IgM. IgA aCL and IgA anti-β2GPI were measured as exploratory markers but were not included in criteria-aPL classification. Patients were categorized according to criteria and non-criteria aPL status. Hierarchical clustering was performed using the 26 solid-phase aPL markers. Continuous outcomes were compared using Kruskal–Wallis or Wilcoxon rank-sum tests, and categorical outcomes using Fisher’s exact test. Birth-weight analyses were restricted to live births. Treatment status was assessed using Fisher’s exact test with Haldane–Anscombe correction for odds ratio estimation when appropriate.

**Results:**

Among live births, gestational age at delivery and birth weight differed significantly across antibody-profile groups. Patients positive for both criteria and non-criteria aPLs had the lowest median gestational age and birth weight, 36.43 weeks (IQR, 34.57–38.00) and 2.15 kg (IQR, 1.88–2.55), respectively. The overall differences were significant for gestational age and birth weight, with p values of 0.010 and 0.007. Hierarchical clustering identified three aPL phenotypes with significant differences in gestational age and birth weight among live births, with p values of 0.011 and 0.018, respectively. In the criteria-aPL-negative and chromosomally normal subgroup, non-criteria aPL positivity was mainly driven by IgM aPE, IgM aPS/PT, and aANXA5. Treatment status was strongly associated with pregnancy outcome.

**Conclusion:**

Comprehensive profiling of non-criteria aPLs may provide additional risk-stratification information in pregnancies complicated by FGR, particularly among patients negative for conventional criteria aPLs. These findings require validation in larger prospective cohorts with standardized antibody testing and treatment protocols.

## Introduction

Antiphospholipid syndrome (APS) is an acquired autoimmune disorder characterized by thrombotic and/or obstetric morbidity in the persistent presence of antiphospholipid antibodies (aPLs).

(1–3). Obstetric manifestations include recurrent pregnancy loss, fetal death, preeclampsia, placental insufficiency, and fetal growth restriction (FGR). Current APS classification criteria include lupus anticoagulant (LA), anticardiolipin antibodies (aCL) IgG/IgM, and anti-β2-glycoprotein I antibodies (anti-β2GPI) IgG/IgM as laboratory criteria (4).

Seronegative APS (SN-APS) has been proposed to describe patients with clinical features suggestive of APS but persistently negative conventional criteria aPLs (5, 6). In obstetric practice, this issue may be relevant to patients with recurrent pregnancy morbidity, fetal death, or placental insufficiency in whom alternative causes are not identified. Because these patients do not meet conventional laboratory criteria, their risk stratification and management remain challenging.

Non-criteria aPLs have been investigated as potential supplementary markers in this context. These include antibodies against phosphatidylethanolamine (aPE), phosphatidylserine/prothrombin complex (aPS/PT), annexin A5 (aANXA5), prothrombin, and other phospholipid or phospholipid-binding protein targets (7). Several studies have reported associations between selected non-criteria aPLs, particularly aPS/PT, and thrombotic or obstetric manifestations (8–12). However, the clinical utility of extended aPL testing remains incompletely established, partly because available studies differ in patient selection, assay platforms, positivity thresholds, and outcome definitions (13, 14).

FGR is a clinically heterogeneous condition and may result from placental insufficiency, maternal disease, fetal abnormalities, infections, or genetic causes. Although APS is recognized as one potential immune-mediated contributor to placental dysfunction, the role of extended non-criteria aPL profiles in pregnancies complicated by FGR remains insufficiently characterized. In particular, few studies have applied data-driven approaches to identify antibody patterns associated with adverse fetal growth outcomes.

Therefore, this retrospective cohort study aimed to evaluate criteria and non-criteria aPL profiles in pregnancies complicated by FGR or related adverse obstetric presentations. We specifically sought to: 1) compare live-birth outcomes across antibody-profile groups; 2) identify immune phenotypes using unsupervised hierarchical clustering of solid-phase aPL markers; 3) describe non-criteria aPL positivity in criteria-aPL-negative and chromosomally normal suspected seronegative APS-like cases; and 4) explore the association between treatment status and pregnancy outcome.

## Materials and methods

### Study design and ethical approval

This retrospective cohort study was conducted to investigate the impact of comprehensive aPLs profiles—encompassing both criteria and non-criteria aPLs—on fetal growth and pregnancy outcomes. The study collected and integrated the clinical baseline characteristics and serological antibody detection data of the patients. This study was approved by the institutional review board of Mianyang Central Hospital (Approval number: S20240229-01). All participants provided written informed consent and were informed about the study’s purpose, in accordance with the Declaration of Helsinki.

### Study population

The study cohort consisted of pregnant women who received prenatal care and delivered or terminated their pregnancies at Mianyang Central Hospital. Patients were eligible if they underwent comprehensive aPL profiling and had complete clinical and pregnancy outcome data. Pregnancies with major non-immune causes of adverse fetal outcomes were not included, including multiple gestations, confirmed fetal chromosomal abnormalities or major structural malformations, severe placental structural abnormalities, and confirmed or strongly suspected severe intrauterine infections. Infectious causes were assessed according to routine obstetric evaluation, including maternal history, ultrasound findings, inflammatory markers where available, and TORCH-related testing when clinically indicated. Detailed inclusion and non-inclusion criteria are provided in the Supplementary materials.

### Antiphospholipid antibody testing

A comprehensive panel of 26 aPL markers was assessed. aCL IgG, IgA, and IgM and anti-β2GPI IgG, IgA, and IgM were measured using enzyme-linked immunosorbent assay kits from Werfen. Anti-β2GPI domain 1 (aβ2GPI-D1) IgG/IgM and anti-annexin A2 IgG/IgM were measured using ELISA kits from Beijing Human Manufacturing. Anti-phosphatidylserine/prothrombin complex IgG/IgM were measured using ELISA kits from Werfen. A 16-item non-criteria phospholipid antibody panel, including IgG and IgM antibodies against phosphatidic acid, phosphatidylglycerol, phosphatidylethanolamine, phosphatidylcholine, phosphatidylinositol, phosphatidylserine, prothrombin, and annexin A5, was assessed using an immunoblot assay from Generic Assays. Lupus anticoagulant was assessed by diluted Russell’s viper venom time using a Werfen coagulation assay kit. LA testing was performed according to the principles of the ISTH guideline, and the testing sequence followed CLSI recommendations.

Positivity was defined according to the manufacturers’ recommended cut-off values. For ELISA-based assays, positivity was defined as >20 units for aCL IgG/IgA/IgM, anti-β2GPI IgG/IgA/IgM, aβ2GPI-D1 IgG/IgM, and anti-annexin A2 IgG/IgM, and >30 units for aPS/PT IgG/IgM. For the immunoblot assay, results were classified as negative or positive according to the manufacturer’s instructions. LA positivity was defined as an LA1/LA2 ratio >1.2. No separate category of strong positivity was predefined in this study. For statistical analyses, antibody results were dichotomized as negative or positive according to these cut-offs. Except for LA, all patients with positive antibody results underwent repeat testing of the positive antibody after at least 12 weeks.

### Variable definitions and antibody profile classification

Gestational age at delivery was converted into continuous decimal weeks. For example, 36 + 4 weeks was converted to 36.57 weeks.

Criteria aPLs were defined as LA, aCL IgG/IgM, and anti-β2GPI IgG/IgM, in accordance with current APS classification criteria. IgA aCL and IgA anti-β2GPI were not considered criteria antibodies and were analyzed only as exploratory markers.

Non-criteria aPLs included aβ2GPI-D1 IgG/IgM, aPS/PT IgG/IgM, aPT IgG/IgM, aPS IgG/IgM, aPI IgG/IgM, aPG IgG/IgM, aPA IgG/IgM, aPE IgG/IgM, aANXA5 IgG/IgM, and aANXA2 IgG/IgM. Patients were categorized into four antibody-profile groups: all negative, non-criteria positive only, criteria positive only, and both criteria and non-criteria positive. The number of positive non-criteria antibodies was also calculated to quantify non-criteria antibody burden.

### Statistical analysis

Continuous variables were summarized as mean ± standard deviation or median [interquartile range], depending on distribution. Categorical variables were summarized as counts and percentages. The primary live-birth outcomes were gestational age at delivery and birth weight. Birth-weight analyses were restricted to live births. Group comparisons for continuous outcomes were performed using the Kruskal–Wallis test for more than two groups and the Wilcoxon rank-sum test for two-group comparisons. Categorical outcomes, including pregnancy loss and preterm birth, were compared using Fisher’s exact test because of the small subgroup sizes.

Hierarchical clustering was performed using binary positivity data from the 26 solid-phase aPL markers. Hierarchical clustering was performed using binary positivity data from the 26 solid-phase aPL markers with Euclidean distance and Ward.D2 linkage. Treatment status was evaluated using Fisher’s exact test for pregnancy outcome. Because no pregnancy-loss events occurred in the treated group, a Haldane–Anscombe corrected odds ratio with 95% confidence interval (CI) was calculated. Treatment-related findings were considered exploratory because treatment allocation was not randomized and the untreated group was small.

All statistical analyses were conducted using R software (Version 4.5.2). *P*-values less than 0.05 were considered statistically significant.

## Results

### Baseline demographic characteristics and distinct pregnancy outcomes stratified by aPL profiles

This retrospective cohort study included a total of 104 pregnant patients who possessed comprehensive clinical records and complete serological data for both criteria and non-criteria aPLs. Based on the comprehensive antibody screening results obtained during the first trimester, the patients were systematically categorized into four distinct immune phenotype groups: the all-negative group (n = 32, 30.8%), the non-criteria only group (n = 40, 38.5%), the criteria only group (n = 10, 9.6%), and the both positive group (n = 22, 21.2%). Baseline demographic and clinical characteristics are summarized in **Table 1**. No statistically significant differences were observed across the four antibody-profile groups in maternal age, number of pregnancies, insulin resistance, hypothyroidism, treatment status, pregnancy loss, preterm birth, or circumvallate placenta. However, residual confounding cannot be excluded because of the retrospective design and limited subgroup sizes.

**Table 1:** Baseline by antibody profile.

Characteristic	All negative	Non-criteria only	Criteria only	Both positive	*P* value
n	32	40	10	22	
Age (years)	31.34 (4.60)	32.12 (4.45)	30.40 (2.80)	30.73 (5.34)	0.583
Insulin resistance (%)	4 (12.5)	5 (12.5)	1 (10.0)	0 (0.0)	0.316
hypothyroidism (%)	8 (25.0)	11 (27.5)	4 (40.0)	8 (36.4)	0.686
Number of pregnancies	2.50 (1.08)	2.48 (1.06)	2.10 (0.99)	2.59 (1.26)	0.704
Treatment (%)	32 (100.0)	37 (92.5)	9 (90.0)	19 (86.4)	0.139
Pregnancy loss (%)	0 (0.0)	3 (7.5)	1 (10.0)	1 (4.5)	0.304
Preterm birth (%)	5 (15.6)	11 (27.5)	2 (20.0)	7 (31.8)	0.491
Circumvallate placenta (%)	7 (21.9)	8 (20.0)	2 (20.0)	11 (50.0)	0.071
Gestational age at delivery (weeks)	38.21 (37.39, 38.64)	37.36 (36.57, 38.46)	38.00 (36.71, 38.54)	36.21 (34.04, 37.96)	0.01

**Figure 1:**
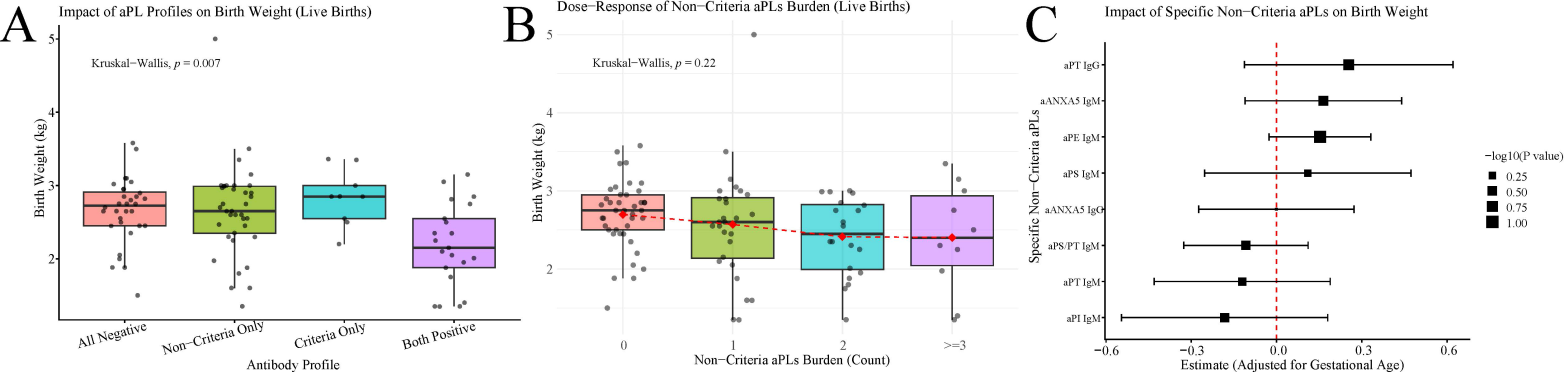
Impact of aPLs profiles on birth weight and the effects of specific non-criteria aPLs. **(A)** Box plots comparing the birth weight of live births across four different aPL antibody profiles (All Negative, Non-Criteria Only, Criteria Only, Both Positive) (Kruskal-Wallis test, *p* = 0.007). **(B)** Dose-response trend between non-criteria aPLs burden (count of positive antibodies: 0, 1, 2, ≥3) and birth weight in live births (Kruskal-Wallis test, *p* = 0.22). **(C)** Forest plot exploratory gestational-age-adjusted associations between selected non-criteria aPLs and birth weight. Only antibodies with sufficient positive cases for model estimation and/or clinically relevant effect estimates are displayed. Antibodies with no or extremely rare positivity were not shown because their estimates were unstable or not estimable. Positive coefficients, including the apparent positive association for aPT IgG, should be interpreted cautiously and do not imply a protective biological effect.

Despite this baseline homogeneity, live-birth outcomes differed across antibody-profile groups. Among live births, gestational age at delivery differed significantly among the groups (*p* = 0.01), with the “both positive” group exhibiting the shortest median gestational age at 36.43 weeks (Interquartile Range [IQR]: 34.57, 38.00). Furthermore, birth weight among live births also differed across groups.

(*p* = 0.007) (**Figure 1A**). As illustrated in **Supplementary Table 1**, the “all-negative” group recorded the highest median birth weight at 2.72 kg (IQR: 2.45, 2.91). Patients positive for either non-criteria or criteria antibodies alone exhibited intermediate birth weights, suggesting a moderate impairment of fetal growth. The both-positive group had the lowest median birth weight, 2.15 kg (IQR: 1.88, 2.55). The overall differences were significant for gestational age and birth weight, with Kruskal–Wallis p values of 0.010 and 0.007, respectively. Preterm birth rates did not differ significantly across antibody-profile groups, although the highest rate was observed in the both-positive group, 33.3%, *p* = 0.481.

### Association of non-criteria aPL burden and specific isotypes with birth weight

To explore whether non-criteria aPL burden was associated with fetal growth, we compared birth weight across burden categories. Birth weight tended to be lower with increasing non-criteria aPL burden, but the overall difference was not statistically significant in birth weight as the number of positive non-criteria antibodies increased from 0 to ≥3 (*p* = 0.22) (**Figure 1B**).

To explore antibody-specific associations with birth weight, we performed regression analyses adjusted for gestational age at delivery. **Figure 1C** presents selected non-criteria aPLs with sufficient positive cases for model estimation and/or clinically relevant effect estimates. Antibodies with no positive cases or extremely rare positivity were not displayed because the corresponding estimates would be unstable and difficult to interpret. Several IgM non-criteria antibodies, including aPS IgM, aANXA5 IgM, aPE IgM, and aPS/PT IgM, showed negative coefficients for birth weight after adjustment for gestational age. However, these antibody-specific findings should be interpreted cautiously because of the small number of positive cases and the exploratory nature of the analysis. The apparent positive coefficient for aPT IgG should also be interpreted with caution, as the number of aPT IgG-positive cases was small and the estimate may reflect sparse-data instability rather than a biologically protective association.

### Unsupervised hierarchical clustering of aPL profiles and live-birth outcomes

**Figure 2:**
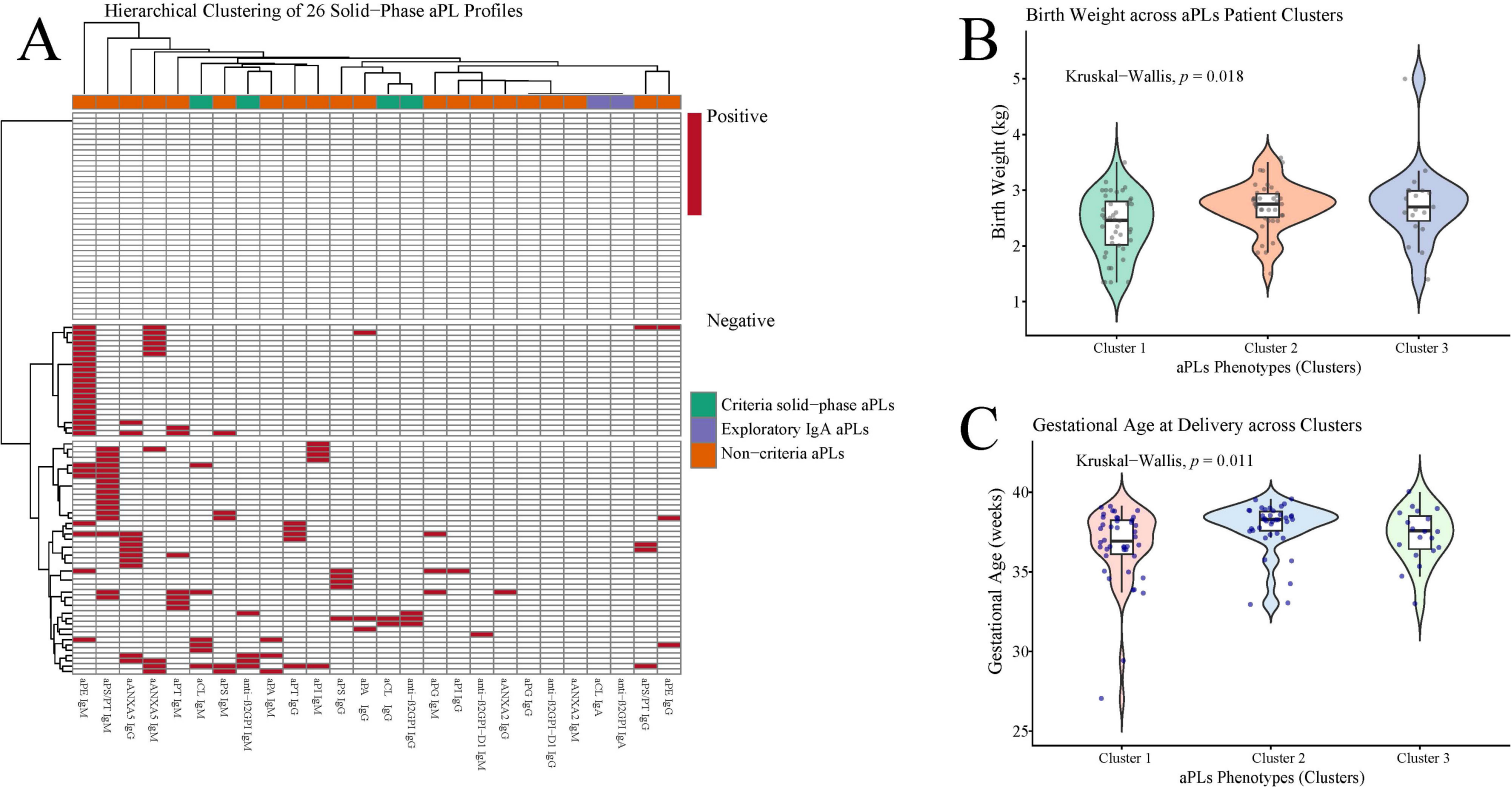
Unsupervised hierarchical clustering of 26 solid-phase aPLs markers and pregnancy outcomes across different serological phenotypes. **(A)** Hierarchical clustering heatmap of 26 solid-phase aPL markers (LA was not included in the clustering matrix), stratifying the patient cohort into three distinct immune phenotype clusters (Cluster 1, 2, and 3). **(B)** Distribution and comparison of birth weight across the three different aPL patient clusters (Kruskal-Wallis test, *p* = 0.018). **(C)** Distribution and comparison of gestational age at delivery across the three different aPL patient clusters (Kruskal-Wallis test, *p* = 0.011).

To explore patterns of antibody co-positivity, we performed unsupervised hierarchical clustering using binary positivity data from 26 solid-phase aPL markers. This analysis identified three clusters with different antibody profiles (**Figure 2A**).

Based on the serological distributions presented in **Supplementary Table 2**, the three clusters were described as antibody-profile phenotypes rather than prespecified risk categories. Cluster 1 (n = 44, 42.3%) showed more frequent multi-antibody positivity, including aPS/PT IgM positivity in 38.6% of patients and aPE IgM positivity in 15.9% of patients. Cluster 2 (n = 39, 37.5%) was characterized by low overall antibody positivity, with most measured solid-phase aPL markers being negative. Cluster 3 (n = 21, 20.2%) was characterized mainly by aPE IgM positivity, which was present in all patients in this cluster, whereas most other markers were negative.

Hierarchical clustering based on 26 solid-phase aPL markers identified three patient clusters with distinct live-birth outcomes. Cluster 1 showed the lowest median gestational age at delivery, 36.93 weeks (IQR, 36.11–38.25), and the lowest median birth weight, 2.46 kg (IQR, 2.02–2.80). Cluster 2 showed the highest median gestational age and birth weight, 38.29 weeks (IQR, 37.57–38.79) and 2.75 kg (IQR, 2.51–2.94), respectively. The between-cluster differences were significant for both gestational age and birth weight, with *p* values of 0.011 and 0.018, respectively. The preterm birth rate was highest in Cluster 1, 35.7%, but the difference did not reach statistical significance, *p* = 0.059 (**Figures 2B, C**, **Supplementary Tables 2**, **3**).

### Subgroup analyses according to maternal metabolic comorbidities

**Figure 3:**
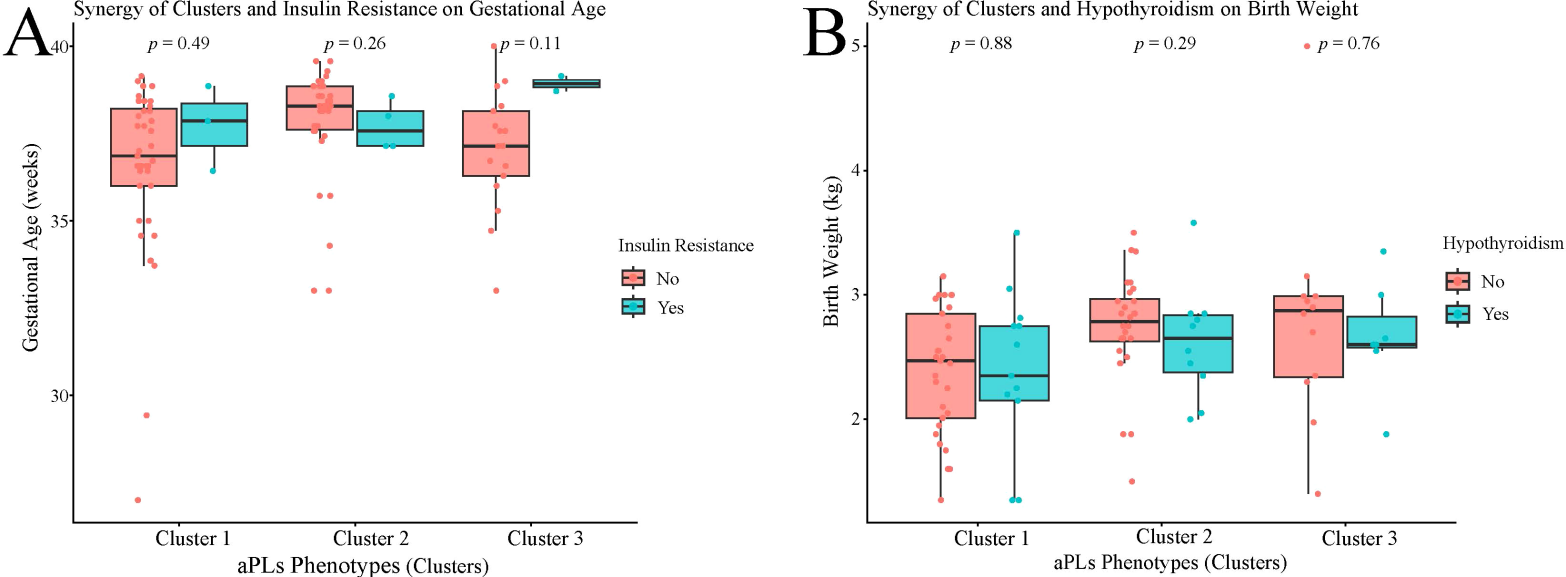
Synergistic effects of aPLs phenotype clusters and maternal metabolic comorbidities on pregnancy outcomes. **(A)** Comparison of gestational age between patients with and without insulin resistance within each aPL phenotype cluster. **(B)** Comparison of birth weight between patients with and without hypothyroidism within each aPL phenotype cluster.

Maternal metabolic disorders are well-established independent risk factors for adverse obstetric outcomes. We explored whether insulin resistance or hypothyroidism modified the association between antibody clusters and live-birth outcomes. Within each cluster, gestational age at delivery did not differ significantly according to insulin resistance status (*p*-values for Clusters 1, 2, and 3 were 0.88, 0.29, and 0.77, respectively). Similarly, birth weight did not differ significantly according to hypothyroidism status (*p*-values for Clusters 1, 2, and 3 were 0.88, 0.29, and 0.77, respectively) (**Figure 3B**). These analyses were exploratory and limited by small subgroup sizes.

### Association between treatment status, pregnancy loss, and live-birth outcomes

A critical aspect of the present cohort was the difference in pharmacological management. Among the 104 patients, 97 received combination treatment consisting of hydroxychloroquine (HCQ), low-dose aspirin (LDA), and low-molecular-weight heparin (LMWH). Among these treated patients, 6 severe cases additionally received intravenous immunoglobulin (IVIG). The remaining 7 patients did not receive these treatments.

Pregnancy loss was more frequent among untreated patients. Specifically, 5 of the 7 untreated patients experienced intrauterine fetal death (IUFD), whereas no pregnancy-loss events occurred in the treated group. Fisher’s exact test showed a significant association between treatment status and pregnancy loss in the full cohort, *p* = 2.28×10^−7^. Because no pregnancy-loss events occurred in the treated group, a Haldane–Anscombe corrected odds ratio was calculated. The corrected odds ratio for pregnancy loss in untreated versus treated patients was 429.0, with a wide 95% CI of 18.28–10067.24. This result should be interpreted cautiously because treatment allocation was not randomized and the untreated group was small.

When pregnancy loss was analyzed across the four antibody-profile groups, the difference was not statistically significant: All Negative, 0.0%; Non-Criteria Only, 7.5%; Criteria Only, 10.0%; and Both Positive, 4.5%; *p* = 0.304 (**Table 1**). This lack of significant difference across antibody profiles should be interpreted in the context of the high proportion of treated patients in all groups.

**Figure 4:**
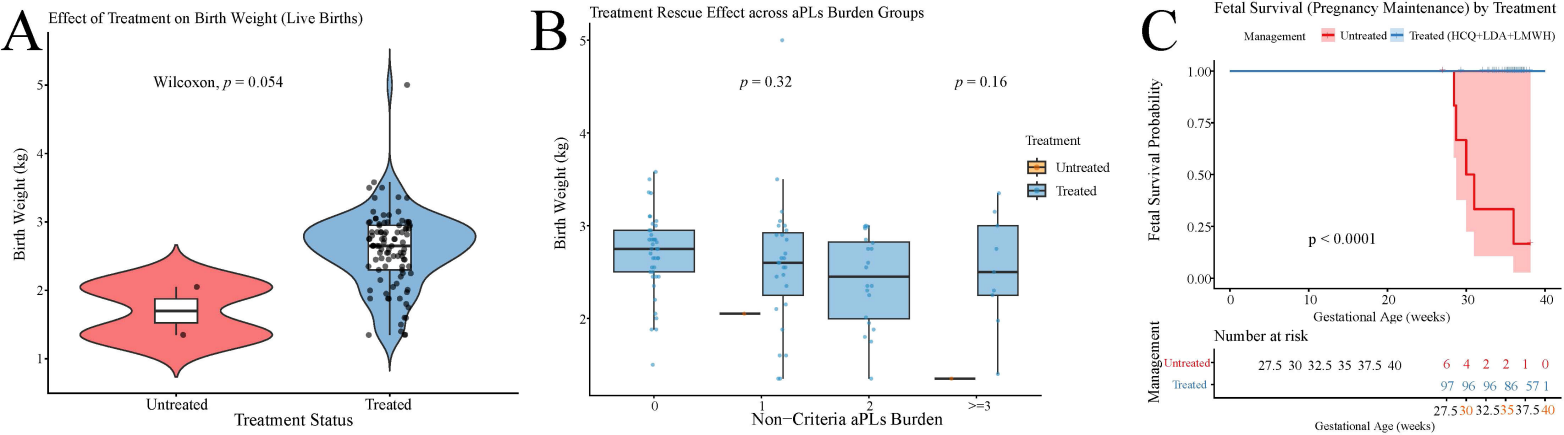
Association between treatment status and pregnancy outcomes. **(A)** Birth weight among live births according to treatment status. **(B)** Birth weight among live births stratified by non-criteria aPL burden and treatment status. **(C)** Kaplan–Meier curves comparing pregnancy maintenance between treated and untreated patients. Treatment-related analyses were exploratory because treatment allocation was not randomized and the untreated group was small.

We further explored the association between treatment status and birth weight among live births. As shown in **Figure 4A**, neonates born to treated mothers had a higher median birth weight than the two surviving neonates in the untreated group, although this difference did not reach conventional statistical significance, *p* = 0.054. This comparison was limited by the very small number of live births in the untreated group. In analyses stratified by non-criteria aPL burden (**Figure 4B**), birth weight appeared relatively stable among treated patients across burden categories. However, comparisons between treated and untreated patients within specific burden strata did not reach statistical significance, including burden 1, *p* = 0.32, and burden ≥3, *p* = 0.16.

Pregnancy maintenance was also assessed using Kaplan–Meier analysis with gestational age at delivery or pregnancy loss as the time variable (**Figure 4C**). The survival curves differed significantly between treated and untreated patients, with a log-rank *p* < 0.0001. However, because of the retrospective design, non-randomized treatment allocation, and small untreated group, this analysis should be considered exploratory. These findings indicate an association between treatment status and pregnancy outcome, but they do not establish treatment efficacy.

### Serological characteristics and clinical implications in the suspected SN-APS cohort

**Figure 5:**
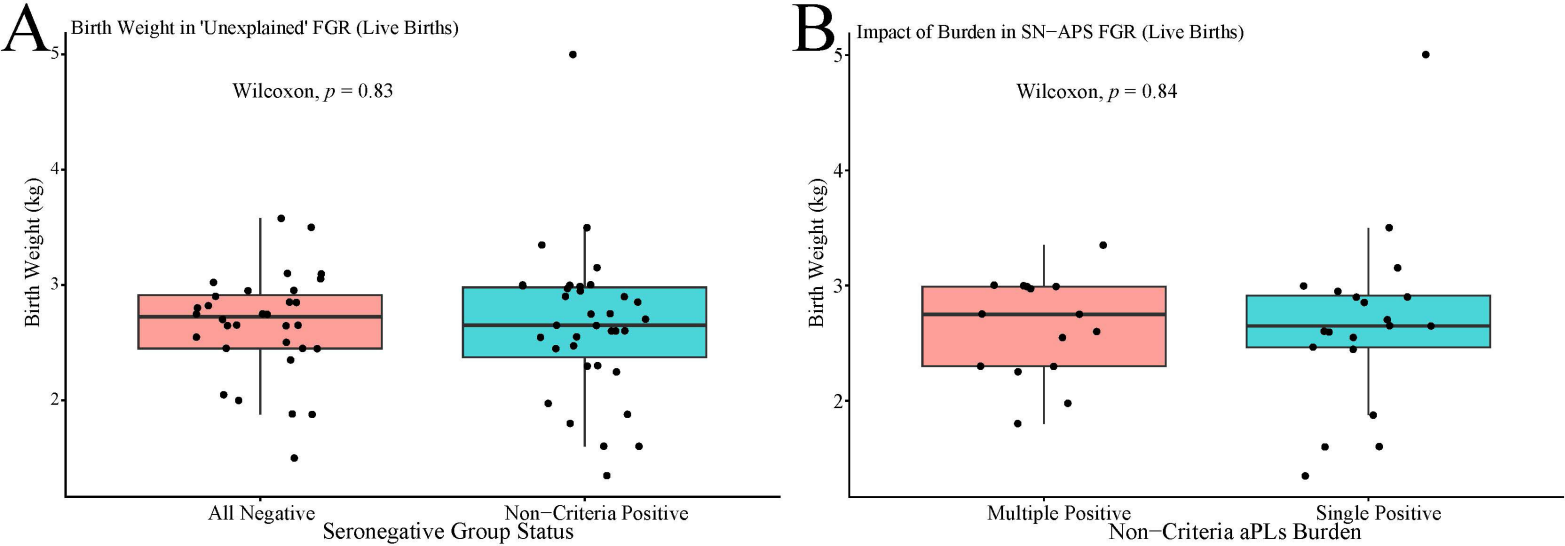
Antibody burden and outcomes in the SN-APS FGR cohort. **(A)** Comparison of birth weight (live births) between the all-negative group and the non-criteria positive group within the “unexplained” FGR cohort (Wilcoxon test, *p* = 0.83). **(B)** Comparison of the impact of multiple positive versus single positive non-criteria aPLs on birth weight in the SN-APS FGR subgroup (Wilcoxon test, *p* = 0.84).

We further examined patients with unexplained FGR or adverse placental events who were negative for criteria aPLs and had no chromosomal abnormalities. In this subgroup, the most frequent non-criteria marker was aPE IgM, with a positivity rate of 28.57%, followed by aPS/PT IgM and aANXA5 (**Supplementary Table 4**). Birth weight did not differ significantly according to overall non-criteria aPL positivity or single versus multiple non-criteria aPL positivity (**Figure 5**). These findings should be interpreted cautiously because of the limited sample size.

## Discussion

This retrospective cohort study evaluated the serological landscape of aPLs in pregnancies complicated by FGR. By incorporating both criteria and non-criteria aPLs and applying unsupervised hierarchical clustering, we aimed to characterize heterogeneous maternal antibody profiles and explore their associations with FGR-related outcomes. Overall, our findings suggest that broader aPL profiling may provide additional information beyond conventional Obstetric APS classification. In particular, the coexistence of criteria and non-criteria aPLs, as well as specific multi-antibody profiles, appeared to be associated with more adverse fetal growth outcomes. However, given the retrospective design and limited sample size in several subgroups, these findings should be interpreted as exploratory and require validation in larger prospective cohorts.

A notable observation from our cohort was that patients positive for both criteria and non-criteria aPLs tended to have less favorable FGR-related outcomes than those with either antibody category alone. The “both positive” group showed lower neonatal birth weights and earlier gestational ages at delivery, suggesting that combined antibody positivity may be associated with a higher-risk phenotype. Previous studies have reported associations between criteria aPLs, particularly anticardiolipin antibodies, and intrauterine growth restriction, including in patients receiving standard treatments (15). Mechanistically, criteria aPLs have been implicated in placental dysfunction through pathways such as disruption of the annexin A5 anticoagulant shield and induction of trophoblast apoptosis (16). It is therefore plausible that the additional presence of non-criteria aPLs could contribute to a broader pattern of placental injury. Nevertheless, our data do not establish causality, and the observed associations should be considered hypothesis-generating.

When non-criteria antibodies were evaluated individually, no single non-criteria aPL consistently showed an independent association with reduced birth weight. In addition, the apparent positive coefficient observed for aPT IgG in the exploratory regression analysis was based on few positive cases and should be regarded as an unstable estimate rather than evidence of a protective effect. This may reflect limited statistical power, heterogeneity among antibody subtypes, or the possibility that non-criteria aPLs act through cumulative or interactive mechanisms rather than through one dominant antibody. Although the progressive decline in neonatal birth weight with increasing non-criteria antibody burden did not reach overall statistical significance in the updated analysis, the observed trend suggests that antibody burden may still have clinical relevance. These findings support the potential value of evaluating non-criteria aPLs as a panel rather than relying exclusively on isolated antibody results. Prior studies have also suggested that extended aPL profiling may help characterize patients with clinical manifestations suggestive of APS but negative for conventional criteria antibodies (17). However, the clinical utility of such testing for FGR risk stratification remains to be confirmed in prospective studies.

To further assess the cumulative and interactive burden of these autoantibodies, we applied unsupervised agglomerative hierarchical clustering and identified three antibody-profile clusters. Compared with simple antibody-count approaches, clustering may better capture patterns of co-positivity and serological heterogeneity. In our cohort, Cluster 1, characterized by a higher frequency of multiple antibody positivity, including aPS/PT IgM and aPE IgM, was associated with less favorable FGR-related outcomes. This observation is broadly consistent with recent studies using cluster-based approaches in aPL-associated adverse pregnancy outcomes, which reported that patients with multi-antibody positivity may have greater placental involvement and higher rates of preterm delivery (18). However, because cluster sizes were limited and treatment exposure was unevenly distributed, these results should not be interpreted as definitive evidence that this cluster independently determines FGR severity.

We also examined whether maternal metabolic conditions modified the association between antibody profiles and FGR outcomes. In subgroup interaction analyses, the associations between high-risk antibody profiles and fetal growth outcomes were not significantly modified by insulin resistance or hypothyroidism. Maternal endocrine and metabolic abnormalities are recognized contributors to altered fetal growth and adverse pregnancy outcomes (19–21). In the present cohort, however, the aPL-related serological profile appeared to remain associated with FGR outcomes irrespective of these metabolic comorbidities. This finding may suggest that immune-mediated placental dysfunction contributes to fetal growth impairment beyond the influence of maternal metabolic status. Nevertheless, the lack of statistically significant interaction should be interpreted cautiously, as subgroup analyses were limited by sample size and may have been underpowered to detect modest effect modification.

Treatment status provided important context for interpreting pregnancy outcomes in this cohort. Most patients received combination therapy with hydroxychloroquine, low-dose aspirin, and low-molecular-weight heparin, whereas only a small minority were untreated. Pregnancy loss was more frequent in the untreated group, and treatment status was significantly associated with pregnancy loss in the full cohort. Specifically, Fisher’s exact test showed a significant association between treatment status and pregnancy loss, and the Haldane–Anscombe corrected odds ratio for pregnancy loss in untreated versus treated patients was large but imprecise, with a wide confidence interval. Because no pregnancy-loss events occurred in the treated group, this estimate should be interpreted with caution. Importantly, treatment allocation was not randomized, the untreated group was small, and treatment decisions may have been influenced by clinical characteristics. Therefore, these findings support an association between treatment status and pregnancy outcome but do not establish treatment efficacy.

The strong imbalance in treatment exposure may also help explain why IUFD rates did not differ significantly across the four baseline antibody-profile groups. If treatment reduced the risk of pregnancy loss among higher-risk patients, differences attributable to baseline serological profiles could have been attenuated. However, this interpretation remains speculative because the study design does not allow separation of treatment effects from baseline risk, clinical decision-making, or other confounders. Similarly, the comparison of birth weight between treated and untreated live births was limited by the very small number of surviving neonates in the untreated group. Although neonates born to treated mothers had a higher median birth weight than the surviving neonates in the untreated group, this difference did not reach conventional statistical significance. Therefore, treatment-related analyses in this study should be considered exploratory and interpreted primarily as contextual information rather than as evidence of therapeutic benefit.

Our study also highlights the potential limitations of relying solely on current classification criteria when evaluating FGR cases with suspected autoimmune involvement. In this cohort, a substantial proportion of patients with otherwise unexplained FGR were positive for non-criteria aPLs. This observation is consistent with previous cross-sectional studies reporting that non-criteria aPLs, particularly aPE and aPS/PT, may be detected in patients with late pregnancy morbidities, including unexplained fetal death and FGR (22). These findings suggest that some cases currently classified as idiopathic FGR may have an autoimmune serological component. However, the presence of non-criteria aPLs alone does not prove causality, and the clinical significance of isolated or low-titer positivity remains uncertain.

The concept of seronegative APS (SN-APS) may be relevant in this context. Patients who are negative for criteria aPLs may still carry other autoantibodies that have been proposed to contribute to APS-like clinical manifestations (23). In particular, aPS/PT has been associated with fetal death in SN-APS cohorts (24). In our cohort, the clinical phenotype, including miscarriage history, birth weight, and preterm birth rate, was not clearly distinguishable between the non-criteria-positive and strictly antibody-negative groups. This finding suggests that non-criteria aPL positivity may help identify a subset of patients with possible autoimmune involvement, but it does not necessarily indicate a more severe FGR phenotype compared with antibody-negative idiopathic FGR. Further studies are needed to determine whether these patients differ in placental pathology, recurrence risk, or response to prophylactic treatment in subsequent pregnancies.

Our sub-analysis of the SN-APS cohort provides additional clinical nuance. Among patients negative for criteria aPLs but positive for non-criteria aPLs, those with a single non-criteria antibody did not show clearly milder FGR outcomes than those with multiple non-criteria antibodies. This observation may suggest that, within otherwise unexplained FGR, the presence of even one non-criteria aPL could be clinically relevant. However, the small size of the SN-APS subgroup limits the strength of this conclusion. Rather than implying equivalence between single and multiple antibody positivity, these results indicate that isolated non-criteria aPL positivity should not be automatically dismissed, particularly when FGR remains otherwise unexplained. Prospective studies with standardized antibody testing, repeated measurements, and placental assessment are needed to clarify the prognostic significance of single versus multiple non-criteria aPL positivity.

The global relevance of APS-related obstetric morbidity should also be considered. Most studies on non-criteria aPLs and obstetric APS have been conducted in Europe, Asia, or North America, whereas data from low- and middle-income regions, including sub-Saharan Africa, remain limited (25, 26). In these settings, adverse pregnancy outcomes such as fetal death and FGR may also be influenced by infectious diseases, hypertensive disorders, malnutrition, delayed access to antenatal care, and limited availability of specialized immunological testing (27). Therefore, the clinical implementation of extended aPL panels requires careful consideration of local epidemiology, laboratory capacity, and competing causes of placental dysfunction. Future multicenter studies including underrepresented regions are needed to determine whether the antibody patterns observed in our cohort are generalizable across diverse populations.

This study has several limitations. First, as a retrospective, single-center study, it is subject to selection bias, referral bias, and residual confounding. External validation in larger, multi-center cohorts is therefore required. Second, the relatively small sample size—particularly within the specific clustered subgroups, the untreated cohort, and the SN-APS cohort—may have limited the statistical power to detect more subtle synergistic effects or differences among the highest antibody burden tiers. Third, our analysis was restricted to serological specimens collected solely at the patients’ initial clinical presentation. The lack of longitudinal assessments of aPL titers throughout the pregnancy prevents us from evaluating the dynamic fluctuations of these autoantibodies and their temporal relationship with fetal growth deceleration. Fourth, the serological evaluation was strictly limited to antiphospholipid antibody profiles. The study did not incorporate other potentially relevant data, such as comprehensive maternal immune cell profiling, genetic screening, or detailed placental pathological examinations, which could provide a holistic view of the underlying mechanisms driving FGR. Fifth, treatment allocation was not randomized, and treatment-related analyses are therefore subject to confounding by indication and should be considered exploratory. Finally, our study primarily focused on immediate perinatal outcomes and lacked long-term neurodevelopmental and metabolic follow-up for the growth-restricted neonates.

In conclusion, comprehensive profiling of criteria and non-criteria aPLs may provide additional information for risk stratification in pregnancies complicated by FGR. Combined criteria and non-criteria aPL positivity and cluster-defined antibody phenotypes were associated with lower gestational age at delivery and lower birth weight among live births. In criteria-aPL-negative and chromosomally normal patients, non-criteria aPL positivity was mainly driven by IgM aPE, IgM aPS/PT, and aANXA5. Treatment status was associated with pregnancy outcome, but this finding should be interpreted cautiously because of the retrospective design, non-randomized treatment allocation, and small untreated group. Larger prospective studies using standardized aPL assays are needed to validate these findings.

## Data Availability

The raw data supporting the conclusions of this article will be made available by the authors, without undue reservation.
