# Supplementary material for: Comprehensive antiphospholipid antibody profiling and unsupervised immune phenotyping in fetal growth restriction
Source: Front Immunol. 2026 Jun 3;17:1845168. doi: 10.3389/fimmu.2026.1845168 (PMC13272371; doi:10.3389/fimmu.2026.1845168)
Supplement: Supplementary file 1 [file Table1.docx]

**Detailed Inclusion and Non-inclusion Criteria**

**Inclusion Criteria:**

1. Pregnant women who received prenatal care and delivered (or terminated pregnancy) at Mianyang Central Hospital.
2. Patients who underwent comprehensive profiling of 26 antiphospholipid antibodies (aPLs), including both criteria and non-criteria aPLs.
3. Patients presenting with adverse pregnancy outcomes or obstetric complications, specifically those with suspected Antiphospholipid Syndrome (APS), Seronegative APS (SN-APS), or diagnosed with unexplained Fetal Growth Restriction (FGR).
4. Availability of complete clinical records, including maternal comorbidities, detailed pharmacological treatment regimens, and definitive pregnancy outcomes.

**Non-inclusion Criteria:**

1. Multiple gestations (e.g., twins, triplets), to avoid inherent confounding effects on birth weight and gestational age.
2. Pregnancies with confirmed fetal chromosomal abnormalities, genetic disorders, or major congenital structural malformations (confirmed via ultrasound or genetic testing).
3. Severe placental structural abnormalities.
4. Patients with evidence of severe intrauterine infection were not included. Infectious causes were assessed according to clinical presentation and routine obstetric evaluation, including maternal history, ultrasound findings, inflammatory markers where available, and TORCH-related testing when clinically indicated. Cases with confirmed or strongly suspected toxoplasmosis, rubella, cytomegalovirus, herpes simplex virus infection, syphilis, or other severe intrauterine infections were not included in the final analysis.
5. Concomitant severe systemic autoimmune diseases (other than secondary APS), severe hepatic or renal dysfunction, or malignancies.

**Supplementary Table 1.** Live Birth Outcomes by Antibody Profile

|  | All Negative | Non-Criteria Only | Criteria Only | Both Positive | *P* value |
| --- | --- | --- | --- | --- | --- |
| n | 32 | 37 | 9 | 21 |  |
| GA_numeric (median [IQR]) | 38.21 [37.39, 38.64] | 37.57 [36.57, 38.57] | 38.29 [37.57, 38.57] | 36.43 [34.57, 38.00] | 0.01 |
| Birth_weight (median [IQR]) | 2.72 [2.45, 2.91] | 2.65 [2.35, 2.99] | 2.85 [2.55, 3.00] | 2.15 [1.88, 2.55] | 0.007 |
| Preterm_birth = 1 (%) | 5 (15.6) | 10 (27.0) | 2 (22.2) | 7 (33.3) | 0.481 |

**Supplementary Table 2.** Cluster Antibody Characteristics of All Patients

|  | Cluster 1 | Cluster 2 | Cluster 3 | *P* value |
| --- | --- | --- | --- | --- |
| n | 44 | 39 | 21 |  |
| Pregnancy loss (%) | 2 (4.5) | 1 (2.6) | 2 (9.5) | 0.429 |
| aCL IgG (positive, %) | 2 (4.5) | 0 (0.0) | 0 (0.0) | 0.68 |
| aCL IgM positive, (%) | 6 (13.6) | 0 (0.0) | 0 (0.0) | 0.015 |
| aCL IgA (positive, %) | 0 (0.0) | 0 (0.0) | 0 (0.0) | NA |
| aβ2GPI IgG (positive, %) | 3 (6.8) | 0 (0.0) | 0 (0.0) | 0.226 |
| aβ2GPI IgM (positive, %) | 4 (9.1) | 0 (0.0) | 0 (0.0) | 0.073 |
| aβ2GPI IgA (positive, %) | 0 (0.0) | 0 (0.0) | 0 (0.0) | NA |
| aANXA2 IgG (positive, %) | 1 (2.3) | 0 (0.0) | 0 (0.0) | 1 |
| aANXA2 IgM (positive, %) | 0 (0.0) | 0 (0.0) | 0 (0.0) | NA |
| aβ2GPI-D1 IgG (positive, %) | 0 (0.0) | 0 (0.0) | 0 (0.0) | NA |
| aβ2GPI-D1 IgM (positive, %) | 1 (2.3) | 0 (0.0) | 0 (0.0) | 1 |
| aPS/PT IgG (positive, %) | 3 (6.8) | 0 (0.0) | 1 (4.8) | 0.252 |
| aPS/PT IgM (positive, %) | 17 (38.6) | 0 (0.0) | 0 (0.0) | <0.001 |
| aPT IgG (positive, %) | 5 (11.4) | 0 (0.0) | 0 (0.0) | 0.034 |
| aPT IgM (positive, %) | 5 (11.4) | 0 (0.0) | 2 (9.5) | 0.079 |
| aPS IgG (positive, %) | 5 (11.4) | 0 (0.0) | 0 (0.0) | 0.034 |
| aPS IgM (positive, %) | 4 (9.1) | 0 (0.0) | 1 (4.8) | 0.174 |
| aPI IgG (positive, %) | 1 (2.3) | 0 (0.0) | 0 (0.0) | 1 |
| aPI IgM (positive, %) | 5 (11.4) | 0 (0.0) | 0 (0.0) | 0.034 |
| aPG IgG (positive, %) | 0 (0.0) | 0 (0.0) | 0 (0.0) | NA |
| aPG IgM (positive, %) | 3 (6.8) | 0 (0.0) | 0 (0.0) | 0.226 |
| aPA IgG (positive, %) | 2 (4.5) | 0 (0.0) | 1 (4.8) | 0.42 |
| aPA IgM (positive, %) | 3 (6.8) | 0 (0.0) | 0 (0.0) | 0.226 |
| aPE IgG (%) | 2 (4.5) | 0 (0.0) | 1 (4.8) | 0.42 |
| aPE IgM (%) | 7 (15.9) | 0 (0.0) | 21 (100.0) | <0.001 |
| aANXA5 IgG (%) | 9 (20.5) | 0 (0.0) | 2 (9.5) | 0.005 |
| aANXA5 IgM (%) | 4 (9.1) | 0 (0.0) | 6 (28.6) | 0.001 |

**Supplementary Table 3.** Cluster Live Birth Outcomes

|  | Cluster 1 | Cluster 2 | Cluster 3 | *P* value |
| --- | --- | --- | --- | --- |
| n | 42 | 38 | 19 |  |
| Gestational age at delivery, weeks (median [IQR]) | 36.93 [36.11, 38.25] | 38.29 [37.57, 38.79] | 37.57 [36.43, 38.50] | 0.011 |
| Birth weight, kg (median [IQR]) | 2.46 [2.02, 2.80] | 2.75 [2.51, 2.94] | 2.70 [2.45, 2.99] | 0.018 |
| Preterm birth = 1 (%) | 15 (35.7) | 5 (13.2) | 4 (21.1) | 0.059 |

**Supplementary Table 4.** Positivity Rates in SN-APS

| Specificity | IgG positivity rate, % | IgM positivity rate, % |
| --- | --- | --- |
| aPS/PT | 1.43 | 10.00 |
| aPT | 1.43 | 7.14 |
| aPS | 5.71 | 5.71 |
| aPI | 1.43 | 2.86 |
| aPG | 0.00 | 1.43 |
| aPA | 1.43 | 1.43 |
| aPE | 1.43 | 28.57 |
| aANXA5 | 8.57 | 8.57 |
| aANXA2 | 0.00 | 0.00 |
| anti-β2GPI-D1 | 0.00 | 0.00 |
